# A systematic review of maternal obesity and breastfeeding intention, initiation and duration

**DOI:** 10.1186/1471-2393-7-9

**Published:** 2007-07-04

**Authors:** Lisa H Amir, Susan Donath

**Affiliations:** 1 Mother & Child Health Research, La Trobe University, Melbourne, Australia; 2 Clinical Epidemiology and Biostatistics Unit, Murdoch Childrens Research Institute, Melbourne, Australia; 3 Department of Paediatrics, University of Melbourne, Australia

## Abstract

**Background:**

Breastfeeding behaviour is multifactorial, and a wide range of socio-cultural and physiological variables impact on a woman's decision and ability to breastfeed successfully. An association has been reported between maternal obesity and low breastfeeding rates. This is of public health concern because obesity is rising in women of reproductive age and the apparent association with increased artificial feeding will lead to a greater risk of obesity in children. The aim of this paper is to examine the relationship between maternal overweight and obesity and breastfeeding intention and initiation and duration.

**Methods:**

A systematic review was conducted in January and February 2007, using the following databases: Medline, CINAHL and the Australian Breastfeeding Association's Lactation Resource Centre. Studies which have examined maternal obesity and infant feeding intention, initiation, duration and delayed onset of lactation were tabulated and summarised.

**Results:**

Studies have found that obese women plan to breastfeed for a shorter period than normal weight women and are less likely to initiate breastfeeding. Of the four studies that examined onset of lactation, three reported a significant relationship between obesity and delayed lactogenesis. Fifteen studies, conducted in the USA, Australia, Denmark, Kuwait and Russia, have examined maternal obesity and duration of breastfeeding. The majority of large studies found that obese women breastfed for a shorter duration than normal weight women, even after adjusting for possible confounding factors.

**Conclusion:**

There is evidence from epidemiological studies that overweight and obese women are less likely to breastfeed than normal weight women. The reasons may be biological or they may be psychological, behavioral and/or cultural. We urgently need qualitative studies from women's perspective to help us understand women in this situation and their infant feeding decisions and behaviour.

## Background

Infants not breastfed have increased risks of ill-health – both short- (e.g. gastrointestinal infections [1]) and long-term (such as diabetes [2]). Recent systematic reviews have shown a dose-dependent association between longer duration of breastfeeding and decrease in the risk of overweight in later life [3,4]. Looking at the population impact, it has been estimated that 13,639 cases of obesity (95%CI 7,838, 19,308) could be prevented in England and Wales over 9 years if all infants were breastfed for at least three months [5].

Obesity is an increasing problem globally: populations in poor countries as well as affluent ones are at risk [6]. Reports of obesity among pregnant women in the USA range from 18.5% to 38.3%, making it one of the most frequent high-risk obstetric situations [7]. A recent Australian study reported that 34% of pregnant women were overweight or obese; overweight/obese women had increased adverse maternal and neonatal outcomes, resulting in increased costs of obstetric care [8]. The increase in maternal obesity is accelerating, and is associated with socio-economic disadvantage [9]. It has been recognised that obesity may track across generations, thus prevention is an urgent priority [10].

In 1992, Rutishauser & Carlin reported a negative relationship between maternal obesity and breastfeeding duration and they stated that this was the first study to investigate the effect of over- rather than under-nutrition on the duration of breastfeeding [11]. Since then, a number of studies have found lower rates of breastfeeding in women who are overweight and obese compared to women of normal weight [12-14]. Some researchers have attributed this to physiological causes, such as delayed lactogenesis ("milk coming in") [15] and lower prolactin response [16]. However, as obese women are more likely to belong to subgroups of women with lower rates of breastfeeding than normal weight women, such as lower socioeconomic status [17] and higher depression [18], it is necessary to adjust for these potential confounding factors.

This is an important public health issue as the increasing incidence of maternal obesity and the apparent association with increased artificial feeding of infants will lead to an increasing risk of obesity in children. The aim of this paper is to examine the relationship between maternal overweight and obesity and breastfeeding intention and initiation and duration.

## Methods

A systematic review was conducted in January and February 2007 using the key words "(obesity OR BMI) AND (breastf* OR lactation OR lactating)". The following databases were searched (all languages, from the start of the database):

• Medline via PubMed (8 February 2007) 767 items, 24 were relevant;

• CINAHL (Cumulative Index to Nursing & Allied Health Literature) (8 February 2007) 103 items, 11 relevant (1 additional);

• Australian Breastfeeding Association's Lactation Resource Centre database was searched for 'obesity' (21 January 2007); 172 items, 10 relevant (3 additional).

Most of the articles found in the databases were about the relationship between breastfeeding and *childhood *obesity and were therefore not relevant to this review. All papers related to maternal overweight and obesity and infant feeding were located and included if appropriate. Papers that were case studies, clinical papers or reviews were not included in the tables [19-28]. Research studies were also identified from the reference lists of included articles, and the authors' literature collection was hand-searched (n = 2230; nine additional studies). Papers which had cited the original Rutishauser and Carlin study were identified (n = 6), but no new papers were revealed. The total number of research articles included in this review is 27.

Five papers were excluded as they did not define overweight or obesity (e.g. presented body mass index (BMI, kg/m^2^) as a continuous variable [29-32] or used vague terms, such as "heavy before becoming pregnant" [33]). However, as there were very few studies on women's infant feeding intention and maternal overweight and obesity, a study which examined women's "weight concerns" was included [34] as this information was also relevant. Thus, 22 papers are included in the tables (27 less 5).

Most studies based their evaluation of maternal obesity on the World Health Organization (WHO) definition of obesity [35]: normal weight BMI<25, overweight BMI 25 ≤ 30, obese BMI > 30, or the US Institute of Medicine (IOM) definition [36]: underweight/normal weight BMI < 26.1, overweight BMI 26.1 – 29.0, obese BMI > 29.0.

The papers have been grouped according to the content of the study and presented in tables alphabetically by the first author. As women's infant feeding intention has been found to be the strongest single predictor of breastfeeding behaviour [37], all studies reporting infant feeding intention and maternal obesity, including those with "vague" definitions, have been included in Table 1 (Included studies on maternal obesity and women's infant feeding intentions).

**Table 1:** Included studies on maternal obesity and women's infant feeding intentions

**Authors, Year of Publication, Country, and Year(s) of Study**	**Participants**	**Definitions**	**Results**
Barnes et al, 1997 [34], Bristol, UK, 1991-112	Birth cohort, (Avon Longitudinal Study of Parents and Children) n = 11,907 Multivariate analysis, n = 8431 for 1^st ^week and n = 8392 for 4 months	Intention: asked at 32 weeks (4 time periods, 4 options for each) Eating Disorder Examination Questionnaire, including 'shape concern' and 'weight concern' items (> 2 indicated marked concern)	*Intention to bf 1 week old infant:* Shape concern normal OR 1.22 (95%CI: 1.10, 1.35) Weight concern normal OR 1.20 (95%CI, 1.07, 1.35) *Intention to bf infant up to 4 months:* Shape concern normal OR 1.30 (95%CI: 1.19, 1.42) Weight concern normal OR 1.26 (95%CI: 1.14, 1.40) Multivariate analysis: *Intention to bf 1 week old infant:* Shape concern normal OR 1.25 (95%CI: 1.09, 1.42) Weight concern normal NS *Intention to bf infant up to 4 months:* Shape concern normal OR 1.26 (95%CI: 1.13, 1.42) Weight concern normal OR 1.16 (95%CI: 1.02, 1.32) (Adjusted for demographic variables, smoking, maternal attitudes to baby)
Foster et al 1996 [41], Manchester, UK, yr of study not stated	Antenatal cohort n = 38	Eating Disorder Examination: Shape Concern (SC). Body Satisfaction Scale: General Satisfaction (GS). Measurement of BMI not mentioned in text	*Bf intention and BMI: *NS *Shape concern:* Intended to bf median 0.29 Intended to formula feed median 1.05 (i.e. higher concern) (p = 0.02) *General Satisfaction:* Intended to bf median 38.5 Intended to formula feed median 47.5 (i.e. higher dissatisfaction) (p = 0.004) *Multivariate analysis: *body shape satisfaction independent predictor of infant feeding intention. (Adjusted for social class, GS, SC and maternal-fetal attachment)
Hilson et al 2004 [42], Cooperstown, NY, USA, 1998	Antenatal cohort Eligibility: intended to bf, singleton infant n = 114	IOM definition of obesity Self-reported height and weight	Planned intention (months, mean, sd) Underweight/normal weight 9.3 (5.7) Overweight 9.8 (3.0) Obese 6.9 (4.6) (p < 0.05)

Bf = breastfeeding, BMI = body mass index, IOM = Institute of Medicine, NS = not significant

The indicators suggested for monitoring breastfeeding have been described as:

• Initiation (the infant's first intake of breast milk)

• Intensity (the degree of exclusiveness of breast milk as the source of nourishment for the infant) and

• Total duration (the total length of time that an infant receives any breast milk at all [38].

Authorities recommend that breastfeeding initiation is defined as "ever breastfed/ever given breast milk" so that infants who only went to the breast once or only received expressed breast milk are included [38]. However, research studies have used a range of definitions, including breastfeeding at hospital discharge [12], breastfeeding at four days [39], feeding in last five feeds in hospital [40]. Table 2 (Included studies on maternal obesity and initiation of breastfeeding) includes the studies which have reported breastfeeding initiation (however defined), and the authors' definition when it varies from "ever breastfed". Where the authors have not presented an odds ratio (OR) for breastfeeding initiation we have calculated one using data from their publication (labelled as "our calculation of overweight/obese women not initiating breastfeeding"). These are unadjusted odds ratios as we did not have the data to adjust for potential confounding factors such as income or method of birth. We have not performed a meta-analysis as the definitions of initiation are inconsistent.

**Table 2:** Included studies on maternal obesity and initiation of breastfeeding

**Authors, Year of Publication, Country, and Year(s) of Study**	**Participants**	**Definition of obesity**	**Results**
Donath & Amir 2000 [13], Australia, 1992–95	1995 National Health Survey Children up to years 4 old n = 2612	BMI calculated from self-reported height and weight at time of interview WHO definition of obesity	% (95% CI) Underweight 89.0 (85.8, 92.2) Normal weight 89.2 (87.4, 91.0) Overweight 86.9 (84.0, 89.9) Obese 82.3 (77.6, 87.0) OR* overweight 1.25 OR* obese 1.78
Grjibovski et al 2005 [43], Severodvinsk, Russia, 1999	Antenatal community-based cohort n = 1078	Pre-pregnancy weight defined as under- normal and over-weight based on "doctor's diagnosis" [82]	Underweight 98.3% Normal 98.7% Overweight 100.0% NS
Hilson et al 1997 [12] Cooperstown, NY, USA, 1992–94	Medical record review Eligibility: intended to bf (= bf at birth), healthy singleton infant n = 1109	IOM definition of obesity BMI calculated from pre-pregnancy weight and height	*Quit bf by hospital discharge 2 d after birth:* Normal 4.3% Overweight 8.9% Obese 12.2% OR* overweight 2.17 OR* obese 3.09 *Not bf at discharge (of women who attempted bf at birth): Odds Ratio* Overweight 2.54 (p < 0.05) Obese 3.65 (p = 0.0007)
Hilson et al 2006 [39] Cooperstown, NY, USA, 1988–97	Expanded previous review of medical records [12] Eligibility: intended to bf (= bf at birth), singleton infant, no contraindications to bf, no diabetes n = 2783	IOM definition of obesity BMI calculated from pre-pregnancy weight and height	*Breastfeeding at 4 days:* Underweight 89.0% Normal 90.1% Overweight 88.4% Obese 82.58% Obese women different from underweight and normal weight women OR* overweight 1.19 OR* obese 1.92
Kugyelka et al 2004 [40] upstate New York, 1999–2000	Medical record review, including paediatric record to 6 months of age, all women of 'Hispanic ethnicity' (n = 235) or 'Black race' (n = 263) Eligibility: healthy mothers (BMI > 19) with healthy single, term infant, who attempted to breastfed	IOM definition BMI calculated from pre-pregnancy weight and height	*Baby put to breast < 2 h:* Hispanic women: Black women Normal 71.8% 75.1% Overweight 66.7% 69.2% Obese 61.5% 63.8% (p < 0.05) (p < 0.05) *Fed formula only during last 5 feeds in hospital:* Hispanic women: Black women: Normal weight 9.6% 5.1% Overweight 12.2% 8.0% Obese 12.4% 6.9% Hispanic women OR* overweight 1.48 OR* obese 1.50 Black women OR* overweight 1.62 OR* obese 1.38 *Multivariate analysis:* Hispanic women: obese OR 1.92 (95% CI 1.20, 3.08) of formula and breast in last 5 feeds before discharge compared to breast only (adjusted for maternal age, education, parity, gestation, birth wt, smoking and birth) Other groups NS
Li et al 2002 [83] USA, 1988–1994	The Third National Health and Nutrition Survey (NHANES III), children aged 2 mo to 6 yrs n = 8765 94% response for these children; 99% data of bf available	BMI calculated from self-reported ht and wt at time of interview. WHO definition of obesity	*Ever breastfed:* Normal 58.1% Overweight 46.4% Obese 44.8% OR* overweight 1.60 OR* obese 1.71
Li et al 2003 [49] USA, 1996–98	Pediatric Nutrition Surveillance System and the Pregnancy Nutrition Surveillance System, children aged < 5 years n = 51,329	BMI calculated from self-reported pre-pregnancy wt IOM definition of obesity	Obese women more likely to never breastfeed (p < 0.01) OR 1.28 estimated from Figure 1
Oddy et al 2006 [44] Australia, 1989–1991	Western Australian Pregnancy Cohort Study. Pregnant women recruited from King Edward Memorial Hospital, Perth, WA n = 1803	BMI calculated from pre-pregnancy weight and height (measured by research midwives) WHO definition of obesity	*Never breastfed:* Normal weight 8.2% Overweight 11.4% Obese women 12.4% NS OR* overweight 1.33 OR* obese 1.47
Scott et al 2006 [45] Australia, 2002–2003	2^nd ^Perth Infant Feeding Study, cohort of women recruited in hospital. n = 587	Measurement of maternal weight and height not reported WHO definition of obesity	*Any breastfeeding at hospital discharge:* Normal weight 95.6% Overweight 91.5% Obese 90.7% OR 0.45 (95% CI 0.19, 1.09) OR* overweight 2.02 OR* obese 2.23 *Exclusive breastfeeding at hospital discharge:* Multivariate analysis, Adjusted OR (95% CI): Normal weight 1 (ref) Overweight 0.50 (0.28, 0.89) Obese 0.63 (0.33, 1.20) (adjusted for maternal age, smoking, marital status, occupation, country of birth, parity, antenatal classes, timing of infant feeding decision, delivery, birth weight, special care nursery, mothers' infant feeding attitude, fathers' infant feeding preference, grandmothers' infant feeding preference, whether grandmother had bf) OR* overweight 2.00 OR* obese 1.59
Sebire et al 2001 [14] UK, 1989–1997	St Mary's Maternity Information system database, North West Thames region n = 325,395	BMI calculated from weight at antenatal booking Normal BMI 20-<25 Moderately obese 25-<30 Very obese BMI > 30 (BMI < 20 = underweight - excluded from study)	*Bf at hospital discharge:* Multivariate analysis, Adjusted OR (99% CI): Normal weight 1 (ref) Mod obese 0.86 (0.84, 0.88) Very obese 0.58 (0.56, 0.60) (adjusted for ethnic group, parity, age, history of hypertension, diabetes) OR* overweight 1.16 OR* obese 1.72

bf = breastfed, BMI = body mass index, ht = height, IOM = Institute of Medicine, mo = month, NS = not significant, OR* = our calculation of overweight and obese women not initiating bf compared with normal weight women, WHO = World Health Organization, wk = week, wt = weight

It has been hypothesised that the onset of lactation occurs later in obese women than other women, therefore all studies which have investigated this are listed in Table 3 (Included studies on maternal obesity and delayed onset of lactation).

**Table 3:** Included studies on maternal obesity and delayed onset of lactation

**Authors, Year of Publication, Country, and Year(s) of Study**	**Participants**	**Definition of obesity**	**Results**
Chapman & Perez-Escamilla 1999 [15] USA 1996–1997	Hartford Hospital, Connecticut Healthy, single, term infant n = 192	Women's bodies were classified as slim, average, heavy or obese	Delayed lactogenesis (> 72 hours) Slim/average build 26.4% Heavy/obese build 52.2% *Multivariate analysis* Heavy/obese build OR 3.2 (95 CI% 1.5, 6.7) (adjusted for birth weight, method of birth, ethnicity, serious medical condition, parity, formula feeding day 2)
Chapman & Perez-Escamilla 2000 [46] USA 1997–1998	Connecticut Healthy mothers with a healthy, single, term infant n = 57	Definition of obesity: at least 2 of 3: 1. BMI at 72 h > 30, 2. subscapular skin fold thickness at 72 h > 33.7 mm (> 85%ile) 3. heavy/obese build on day 1.	Multivariate analysis *Onset -Milk transfer at 60 h (< or > 9.2 g/feed)* Obese: OR 6.14 (95%CI: 1.10, 37.41, p = 0.05) compared to non-obese *Onset – Maternal perception (< or > 72 h)* Obese: OR 1.97 (95%CI: 0.29, 13.41, p = 0.49) compared to non-obese *Non-obese:* Women who bf more frequently had higher milk transfer values and earlier onset of lactogenesis, than women who bf less frequently *Obese:* No relationship between these variables
Dewey et al 2003 [47] USA 1999	Davis, California Healthy, single, term infants, planning to bf > 1 m n = 280	BMI measured 2 weeks postpartum BMI > 27.0 taken as overweight/obese	*Delayed lactogenesis (> 72 hours)* Normal 16% Overweight/obese 33% p < 0.05 *Multivariate analysis for delayed lactogenesis:* Overweight/obese: RR 2.46 (95%CI: 1.45, 3.64) (adjusted for C-section, parity, flat nipples, birth weight): *Multivariate analysis for suboptimal infant feeding behaviour on day 7:* Overweight/obese: RR 2.58 (95%CI: 1.07, 5.22).
Hilson et al 2004 [42] USA 1998	Bassett Hospital, Cooperstown, NY Intended to bf, singleton infant n = 114	BMI calculated from pre-pregnancy weight and height IOM definition of obesity	Delayed lactogenesis (> 72 hours) Normal 18.5% Overweight 30.8% Obese 33.3% Univariate analysis NS Multivariate analysis NS {not enough power to show a difference}
Rasmussen et al 2004 [16] USA Years of study not stated	Bassett Healthcare, Cooperstown, NY n = 40	Pre-pregnancy BMI from medical records IOM definition of obesity	*Duration of feed at 7 days postpartum:* Overweight/obese women: infants fed for longer: 23.2 (sd 5.6) mins, compared to 15.3 (sd 6.1) mins for normal weight women (p < 0.005) *Prolactin response to suckling (ng/ml):* *48 hours* Normal women 26.0 (sd 61.5) Overweight/obese women -10.3 (sd 28.3) p < 0.05 *Prolactin response to suckling (ng/ml):* *7 days* Normal women 80.9 (sd 67.6) Overweight/obese women 57.1 (sd 60.2) NS Other hormones (insulin, estradiol, progesterone): NS difference between groups. However, insulin levels were 44% higher in overweight/obese women at 7 days (non-fasting levels and inadequate power). Path analysis: effect of pre-pregnant BMI on prolactin response at 7 days: -30.9 ng/ml.

The final table (Table 4. Included studies on maternal obesity and duration of breastfeeding) includes studies which report total duration of breastfeeding and, where reported, exclusive breastfeeding; multivariate analysis has been included when this has been conducted.

**Table 4:** Included studies on maternal obesity and duration of breastfeeding

**Authors, Year of Publication, Country, and Year(s) of Study**	**Participants**	**Definition of obesity**	**Results**
Amine et al 1989 [51] Kuwait, Year of study not given	Multistage, stratified sample, mothers of children < 3 years old n = 2833	Height and weight recorded at interview Results expressed as % of reference standard weight for height (Nutrition Institute in Cairo, Egypt)	Mean duration of breastfeeding (month): Weight as % reference median: 80% 4.48 (sd 2.3) 85–119% 5.46 (sd 3.1) 120% 6.36 (sd 3.6)
Baker 2004 [50] Denmark, 1996 onwards	National Birth Cohort Excluded infants <2500 g, <37w gestation, illnesses or conditions expected to negatively affect growth, mother <18y, never breastfeed, mother diabetic n = 3768	BMI calculated from pre-pregnant weight and height WHO definition of obesity	*Full breastfeeding* Underweight 15.5 wk Normal weight 16.3 wk Overweight 15.6 wk Obese 14.9 wk *Any breastfeeding* Underweight 29.5 wk Normal weight 31.3 wk Overweight 29.2 wk Obese 27.3 wk NS
Chapman & Perez-Escamilla 2000 [46] USA, 1997–1998	Connecticut Healthy mothers with a healthy, single, term infant, Caesarean section n = 57	Definition of obesity: at least 2 of 3: 1. BMI at 72 h > 30, 2. subscapular skin fold thickness at 72 h > 33.7 mm (> 85%ile) 3. heavy/obese build on day 1	Multivariate analysis, likelihood of not bf: Non-obese: OR 2.28 (95%CI: 1.02, 5.11) compared with obese women (adjusted for maternal intention, milk transfer and other variables, Table 3, model 1) {Sample too small for multivariate analysis}
Donath & Amir 2000 [13] Australia, 1992–1995	National Health Survey, 1995 Children up to 4 years old Multivariate analysis: n = 1991	BMI calculated at time of interview WHO definition of obesity	Mean duration % (95% CI) Normal 28.7 (27.7, 29.8) Overweight 26.1 (24.3, 28.0) Obese 22.7 (20.1, 25.2) *Multivariate analysis* Normal 1 Overweight 1.15 (1.01, 1.31) Obese 1.36 (1.15, 1.61) p < 0.05 (adjusted for maternal education, marital status, low income, home ownership)
Forster et al 2006 [48] Australia, 1999–2001	Cohort of public patients, Melbourne n = 764	BMI calculated from self-reported height and weight WHO definition of obesity	*Any breastfeeding at 6 months:* Underweight 60.0% Normal 57.0% Overweight 51.9% Obese 37.2% *Multivariate analysis*: OR (95% CI) Underweight 1.15 (0.70, 1.88) Normal 1 Overweight 0.70 (0.43, 1.12) Obese 0.49 (0.28, 0.85) (adjusted for intention, breastfed as a baby, maternal age, smoking, region of birth, attended childbirth education, had formula in hospital, maternal anxiety/depression)
Grjibovski et al 2005 [43] Russia, 1999	Community-based cohort, all pregnant women at antenatal clinics, Severodvinsk n = 1078	Pre-pregnancy weight Defined as under-, normal and over-weight based on "doctor's diagnosis" [82]	*Median duration (months, 25th, 75*^th ^*percentile):* Underweight 5.50 (3.00, 12.00) Normal 5.00 (3.00, 9.00) Overweight 4.25 (2.00, 8.00) NS *Multivariate analysis *NS
Hilson et al 1997 [12] USA, 1992–1994	Medical record review. Bassett Hospital, Cooperstown, NY Healthy singleton infant n = 1109	BMI calculated from pre-pregnancy weight and height IOM definition of obesity	*Exclusive breastfeeding:* Proportional hazards regression: Overweight RR 1.42, p < 0.04 Obese 1.43, p < 0.02 *Any breastfeeding:* Proportional hazards regression: Overweight RR 1.68, p < 0.006 Obese 1.73, p < 0.001 (adjusted for maternal age, smoking, education, gestation, WIC, parity, birth weight, C. section, diabetes)
Hilson et al 2004 [42] USA, 1998	Bassett Hospital, Cooperstown, NY Intended to bf, singleton infant. n = 114	BMI calculated from pre-pregnancy weight and height IOM definition of obesity	*Exclusive breastfeeding *(wks, mean, sd) Underweight/normal 3.6 (3.9) Overweight 2.6 (3.2) Obese 2.7 (2.3) *Any breastfeeding *(wks, mean, sd) Underweight/normal 7.3 (8.9) Overweight 5.6 (5.4) Obese 4.6 (4.6) RR discontinuing bf: obese 2.43 (95%CI: 1.40, 4.20, p = 0.002) cf to underweight/normal wt women Exclusive bf: NS *Multivariate analysis: *RR = 2.03 (95%CI: 1.07, 4.5, p = 0.03) (adjusted for infant feeding intention, work/school, satisfaction with appearance, indifference to bf)
Hilson et al 2006 [39] USA, 1988–1997	Expanded previous review of medical records [12]. Bassett Hospital, Cooperstown, NY Intended to bf, singleton infant. No contraindications to bf, no diabetes. n = 2783	BMI calculated from pre-pregnancy weight and height IOM definition of obesity EBF = last time mother feed only breast milk, without adding non human milk, juice, solids ABF = last feeding of any breast milk to infant	*Median duration of EBF (*wks)*:* Underweight 1.7 Normal 2.0 Overweight 1.7 Obese 1.1 p < 0.05 *Median duration of ABF (*wks): Underweight 8.0 Normal 8.0 Overweight 7.0 Obese 2.0 p < 0.05 *Multivariate analysis*: HR of stopping bf: Obese 1.50 (95%CI 1.11, 2.03) for normal wt gain in pregnancy (adjusted for education, smoking, maternal age, parity, WIC, birth)
Kugyelka et al 2004 [40] USA, Hispanic women: 1998–2000; Black women: 1999–2000	Medical record review, upstate New York, all women of 'Hispanic ethnicity' (mainly Puerto Rican) (n = 235) or 'Black race' (n = 263) Healthy mothers who attempted to breastfeed with healthy single, term infant	BMI calculated from pre-pregnancy height and weight recorded on New York State prenatal form (could be measured or self-reported) IOM definition of obesity EBF = last time mother feed only breast milk, without adding non human milk ABF = last feeding of any breast milk to infant	*Hispanic women:* Obesity assoc with shorter duration of EBF (RR: 1.5; 95%CI: 1.1, 2.0) and ABF (RR: 1.6; 95%CI: 1.1, 2.1) compared to normal wt women *Black women:* No effect of BMI on duration of EBF or ABF
Li et al 2002 [83] USA 1988–1994 (exclusive bf: Phase II, 1991–1994)	The Third National Health and Nutrition Survey (NHANES III) n = 7712	BMI calculated from self-reported ht and wt at time of interview WHO definition of obesity	*Exclusive breastfeeding at 2 months:* Normal 35.4% Overweight 28.2% Obese 25.9% *Breastfeeding at 6 months:* Normal 25.0% Overweight 17.3% Obese 16.9% *Breastfeeding at 12 months:* Normal 10.0% Overweight 5.7% Obese 5.6%
Li et al 2003 [49] USA 1996–1998	Pediatric Nutrition Surveillance System and the Pregnancy Nutrition Surveillance System Children aged < 5 years n = 124,151 (n for multivariate analysis of women who initiated breastfeeding = 13,234)	BMI calculated from self-reported pre-pregnancy weight IOM definition of obesity	*Adjusted breastfeeding duration (weeks):* Underweight 13.3 Normal weight 13.6 Overweight 13.1 Obese 11.8 (p < 0.01) (adjusted for gestational weight gain, birth weight, gestation, parity, maternal age, education, marital status, race, smoking, prenatal care, poverty-income ratio)
Oddy et al 2006 [44] Australia 1989–1991	Western Australian Pregnancy Cohort Study. Antenatal cohort, King Edward Memorial Hospital, Perth, WA n = 1803	BMI calculated from pre-pregnancy weight and height (measured by research midwives) WHO definition of obesity	*Breastfeeding < 2 months:* Normal weight 24.0% Overweight 33.6% Obese women 41.6% p < 0.0005 *Breastfeeding < 4 months:* Normal weight 37.9% Overweight 50.2% Obese women 57.5% p < 0.0005 *Breastfeeding < 6 months:* Normal weight 49.0% Overweight 59.7% Obese women 62.8% p = 0.001 Multivariate Cox hazards regression model: HR (adj) = 1.18 (95%CI 1.05, 1.34) for breastfeeding per month (adjusted for education, maternal age, pregnancy problems, older siblings, smoking, solids before 4 months).
Rutishauser & Carlin 1992 [11] Australia 1984–1985	Primiparas breastfeeding > 14 days Barwon region, Victoria n = 739 (N for multivariate analysis between 570 and 600)	BMI calculated from maternal ht and wt recorded at 1 month postpartum Normal = BMI < 26, Above normal = BMI > 26	*Duration of breastfeeding *associated with BMI (p < 0.05) *Multivariate analysis *(Cox proportional hazards): HR 1.50 (95%CI 1.11, 2.04) (adjusted for smoking, maternal age, time to first breastfeed)
Scott et al 2006 [84] Australia 2002–2003	2^nd ^Perth Infant Feeding Study, cohort of women recruited in hospital. n = 587	Measurement of maternal weight and height not reported WHO definition of obesity	*Any breastfeeding at 6 months *(other time periods also given): Normal 49.0, sd 5.2 Overweight 48.3, sd 9.5 Obese: 35.7, sd 10.1 p < 0.05 *Multivariate analysis *NS

bf = breastfed, BMI = body mass index, ht = height, IOM = Institute of Medicine, mo = month, NS = not significant, WHO = World Health Organization, wk = week, wt = weight

## Results

There were three studies that examined pregnant women's body mass index [41,42] or "weight concerns" [34] and their infant feeding intentions (Table 1. Included studies on maternal obesity and women's infant feeding intentions). In a large population-based study in the UK, women identified as having "marked concern" about body shape and weight on a questionnaire were significantly less likely to intend to breastfeed their infant up to four months after adjusting for a range of variables [34]. A small US study found that obese women planned to breastfeed for a shorter duration (6.9 months) than other women (9.3 to 9.8 months) [42].

Nine of the ten studies of breastfeeding initiation found that overweight and obese women were less likely to commence breastfeeding (Table 2. Included studies on maternal obesity and initiation of breastfeeding). The exception was one study in Russia where virtually all women initiated breastfeeding [43]; the other studies were conducted in the USA (n = 5), Australia (n = 3) and the UK (n = 1). The difference was statistically significant in most studies, but not for black women in the US in the study by Kugyelka [40], nor for women in two studies in Western Australia [44,45]. The estimated size of the effect (OR of not commencing compared with normal weight women) ranged from 1.19 to 2.17 for overweight women and from 1.38 to 3.09 for obese women in these studies (see Table 2).

Table 3 shows the five studies which have examined the relationship between obesity and a delayed onset of lactogenesis (the arrival of a copious milk supply). All studies were conducted in the USA and the sample size ranged from 40 to 280. Delayed onset was found in three studies [15,46,47]. Overweight/obese women were more likely to have late arrival of milk (33%) than normal women (16%), with a relative risk of 2.46 on multivariate analysis [47]. Infants of overweight/obese women were more likely to have suboptimal feeding behaviour on multivariate analysis (RR 2.58) [47]. One study found that overweight/obese women fed their infants for longer (23 minutes) than normal weight women (15 minutes) and had a lower prolactin response to suckling at 48 hours, but not 7 days, compared to normal weight women [16]. One study didn't have enough power to detect a difference [42]. No study found a faster onset of lactation or improved infant feeding in overweight or obese women.

A medical record review in the US found that obese women were less likely to have put the baby to the breast within the first two hours than normal weight women [40].

The studies reporting on maternal obesity and duration of breastfeeding are presented in Table 4. There were fifteen studies, of which seven were conducted in the USA, five in Australia, and one each in Denmark, Kuwait and Russia. The majority of large studies found that obese women breastfed for a shorter duration than normal weight women, even after adjusting for possible confounding factors [11-13,39,44,48,49]. Studies in Russia [43] and Denmark [50] with high breastfeeding initiation rates found no difference in breastfeeding duration according to maternal obesity. A recent Australian study of 764 women found that obese women were less likely to be breastfeeding at six months than women with a normal BMI [48]. Obese women had an odds of 0.49 (95%CI 0.28, 0.85) for breastfeeding at six months compared to women with a normal BMI, adjusted for a range of factors including infant feeding intention, maternal age, smoking and depression [48]. In the USA, Kugyelka and colleagues found no effect of obesity in duration of breastfeeding in black women (while they did find a relationship in Hispanic women) [40]. Only one study, in Kuwait, found that higher maternal weight (120% of standard reference weight for height) was associated with longer duration of breastfeeding [51].

## Discussion

### Possible reasons why overweight/obese women are less likely to breastfeed

#### 1. Anatomical/physiological

Several studies have investigated delayed lactogenesis II (the onset of a copious milk supply) in obese women (Table 3). They reported delayed lactogenesis according to maternal perception and to physiological markers. Obesity remained associated with delayed lactogenesis after adjusting for several possible confounding factors, but infant feeding intention was not included. As obese women intend to breastfeed for shorter durations than other women, perhaps part of the delay in time to first feed [40] and tendency to give up before hospital discharge is behavioural rather than physiological.

Adipose tissue acts as a reservoir for steroid hormones and is also a site of steroid production and metabolism [52,53]. One theory for the delay in lactogenesis II is that progesterone stored in adipose tissue leads to higher progesterone levels in obese women than normal-weight women which disrupts the usual sudden drop in progesterone leading to lactogenesis II [54]. However the only study to investigate this found no difference in serum progesterone levels between obese/overweight women and normal weight women [16].

Although women with large breasts are not necessarily obese, obese women will often have large breasts, and there are indications in the literature that large breasts have been associated with breastfeeding difficulties. Historically it was thought that wet nurses with large breasts were poor milk producers [55]. "Overly large breasts usually betrayed a true poverty of milk, for the heavy fat parts impeded the separation of the milk and its free passage through the narrow conduits to the nipples" [[55], p52]. A study of perceived insufficient milk found that women with a high BMI were more likely to experience an earlier onset of "insufficient milk" (p < 0.05), but this was not significant in multivariate survival analysis [56]. In contrast, Rutishauser and Carlin found that overweight/obese women were *less *likely to give "poor milk supply" as the reason for early cessation of breastfeeding than women of normal weight (p < 0.05) [11].

Women with large breasts may have practical/mechanical difficulties with attaching the baby to the breast [57,58]. It can be awkward to support a large breast while assisting a baby to latch on; sometimes the nipple/areola may not be visible to the mother. Some women with large breasts have broad areolae (rather than conical) with short nipples making it difficult to attach the baby [19]. Lactation consultants have noticed that the weight of a large, heavy breast on the infant's chest can interfere with successful attachment [21].

#### 2. Medical conditions

Obese and overweight women are over-represented in gynaecological and reproductive medicine clinics [53]. They are more likely to have medical conditions such as polycystic ovary syndrome (PCOS) and diabetes, and to experience obstetric complications and caesarean birth than women of normal weight [28,59]. Women with diabetes and those who give birth by Caesarean section may be more likely to experience delayed lactogenesis or low milk supply [60-62]. Some women with PCOS have insufficient milk supply, which is thought to be related to the endocrinological changes associated with the syndrome (high levels of androgens, insulin resistance, frequently low progesterone levels) [23].

Some studies have taken this into account by recruiting only women without medical conditions [30,39] or using multivariate analysis to adjust for these factors [12]. Studies have found that women with early-onset obesity (eg prior to menarche) are more likely to have ovulatory disturbances than women with later-onset obesity [52]. Animal studies have also found that early-onset obesity may negatively affect adult function. Cows with high rates of growth before puberty have less mammary development (as measured by mammary DNA) [63,64]. A meta-analysis of eight experimental studies of prepubertal weight gain in Holstein heifers, found that first-lactation production increased as weight gains increased up to 799 g/day, however higher weight gains were associated with lower milk production [65]. In humans, breastfeeding success (or duration) has not been studied in relation to the onset of obesity in the mother (i.e. in childhood before the development of the breasts) or in later life

#### 3. Socio-cultural

Women who are obese are more likely to belong to social groups who are less likely to breastfeed, such as lower socio-economic status [66,67] and less likely to have been breastfed themselves [31,68]. As with women who smoke, obese women have lower intention to breastfeed [37,69]. Obese women are less likely to participate in preventative health behaviours such having Pap smears and mammography [70]. This may relate to their health beliefs or to feelings of embarrassment with exposure of body parts; it is likely that overweight/obese women may feel more uncomfortable with the idea of breastfeeding in public. Furthermore, large breasts may make if difficult to breastfeed "discretely" and thus "modesty" may another reason for some women to avoid breastfeeding.

Yet in some cultures, maternal weight appears to have no relationship with infant feeding. Indigenous women in Canada have high levels of overweight and obesity and high levels of breastfeeding [71].

#### 4. Psychological

Obese women tend to have greater body image dissatisfaction compared with non-obese women [72]. Women with increased concern about their body shape or weight are less likely to intend to breastfeed [34].

Obese women tend to have lower self-esteem [73] and poorer mental health than normal weight women [74]. Obese mothers are more likely to have postpartum depression [18]; depressed mothers are less likely to continue breastfeeding than non-depressed mothers [75]. A small study of obese formula-feeding mothers found that they spent less time interacting with infants over a 24 hour testing period than non-obese mothers [76].

### What to do about it?

Clinicians need to be aware that obese women are at high risk of not breastfeeding, yet a recent study found clinicians did not manage obese women differently from normal weight women [26]. A new review of maternal obesity in pregnancy acknowledged "the increased risk of lactation failure and delay in establishing lactation postdelivery" [[77] p1137], yet did not mention infant feeding in their management guidelines. Obese women may experience a delay in the onset of lactation but in supportive environments breastfeeding can be successfully established.

Future physiological and epidemiological studies could focus on obese women with a strong intention to breastfeed and without medical or obstetric complications in order to compare breastfeeding success in these women with similar women with normal BMI. To date, no studies have examined this issue from the women's perspective. We urgently need qualitative studies to help us understand obese women and their infant feeding decisions and behaviour. Any potential interventions aimed at helping overweight and obese women to breastfeed successfully need to be evaluated in randomised controlled trials.

## Conclusion

Breastfeeding behaviour is multifactorial, and a wide range of socio-cultural and physiological variables impact on a woman's decision and ability to breastfeed successfully. Breastfeeding rates vary from population group to group – the variation is usually due to social rather than biological factors. Our analysis of maternal smoking and breastfeeding found that maternal infant feeding intention was a more powerful predictor of breastfeeding duration than whether the mother smoked or not [78]. Smokers with a strong intention to breastfeed were more likely to continue breastfeeding that non-smokers with a low intention to breastfeed, i.e. the social factors were more important than the possible negative physiological effects of nicotine on breast milk supply [78].

Evidence suggests that lactational performance is not compromised by low BMI [79]; it is still unclear if obesity per se has a role in reduced lactation in overweight and obese women. There are many psychological, behavioral and cultural reasons that may be responsible for reduced lactation in obese women. It is clear that there is a relationship between obesity and variables associated with lower rates of breastfeeding: lower income, depression, body image concerns. Evidence such as obese women's lower intention [34,41,42] and a 10% greater chance of not putting the baby to the breast in the first two hours of life [40] will lead to physiological differences between obese women and normal weight women – but the differences may not be due to obesity per se.

However a number of epidemiological [11-13,39,44,48,49] and animal studies [80,81] do suggest that maternal obesity is detrimental to lactation. One possibility is that the impact of obesity on lactation is related to the age of development of obesity, as prepubertal obesity is detrimental to lactation in dairy cows [65]. Further studies into the timing of obesity during women's reproductive lifetime may help to clarify this. In addition, qualitative studies as well as quantitative studies should be undertaken to explore the relationship between maternal obesity and breastfeeding.

## Competing interests

The author(s) declare that they have no competing interests.

## Authors' contributions

LHA reviewed the literature and wrote the first draft of the paper. SD calculated summary statistics and revised the paper.

## Pre-publication history

The pre-publication history for this paper can be accessed here:


